# Measuring elimination of podoconiosis, endemicity classifications, case definition and targets: an international Delphi exercise

**DOI:** 10.1093/inthealth/ihv043

**Published:** 2015-07-16

**Authors:** Kebede Deribe, Samuel Wanji, Oumer Shafi, Edridah Muheki Tukahebwa, Irenee Umulisa, Gail Davey

**Affiliations:** a Brighton and Sussex Medical School, Falmer, Brighton, UK; b School of Public Health, Addis Ababa University, Addis Ababa, Ethiopia; c Research Foundation for Tropical Diseases and Environment, Buea, Cameroon; d Department of Microbiology and Parasitology , University of Buea, Buea, Cameroon; e Federal Ministry of Health, Addis Ababa, Ethiopia; f Vector Control Division, Ministry of Health, Kampala, Uganda; g Malaria and OPD Division, Rwanda Biomedical Center, Kigali, Rwanda

**Keywords:** Delphi, Elephantiasis, Elimination, Endemicity, Non-filarial elephantiasis, Podoconiosis

## Abstract

**Background:**

Podoconiosis is one of the major causes of lymphoedema in the tropics. Nonetheless, currently there are no endemicity classifications or elimination targets to monitor the effects of interventions. This study aimed at establishing case definitions and indicators that can be used to assess endemicity, elimination and clinical outcomes of podoconiosis.

**Methods:**

This paper describes the result of a Delphi technique used among 28 experts. A questionnaire outlining possible case definitions, endemicity classifications, elimination targets and clinical outcomes was developed. The questionnaire was distributed to experts working on podoconiosis and other neglected tropical diseases in two rounds. The experts rated the importance of case definitions, endemic classifications, elimination targets and the clinical outcome measures. Median and mode were used to describe the central tendency of expert responses. The coefficient of variation was used to describe the dispersals of expert responses.

**Results:**

Consensus on definitions and indicators for assessing endemicity, elimination and clinical outcomes of podoconiosis directed at policy makers and health workers was achieved following the two rounds of Delphi approach among the experts.

**Conclusions:**

Based on the two Delphi rounds we discuss potential indicators and endemicity classification of this disabling disease, and the ongoing challenges to its elimination in countries with the highest prevalence. Consensus will help to increase effectiveness of podoconiosis elimination efforts and ensure comparability of outcome data.

## Introduction

The possibility of eradicating and eliminating diseases has been discussed over several decades.^1,2^ Historically, several diseases have been targeted for elimination or eradication, including yellow fever, yaws, malaria and leprosy.^3^ Smallpox has been eradicated, while polio and guinea worm are in the end stages.^4^ The London Declaration on neglected tropical diseases includes ambitious goals to eradicate guinea worm disease, and to eliminate lymphatic filariasis, leprosy, sleeping sickness (human African trypanosomiasis) and blinding trachoma by 2020.^5^

Establishing elimination targets and endemicity classifications is vital for creating a standardized monitoring framework, allowing effective communication and ultimately ascertaining elimination of disease. Defining these targets and the associated metrics is fundamental to creating global consensus on disease control across endemic countries. Podoconiosis is a geochemical, non-filarial elephantiasis often occurring in barefoot subsistence farmers who are in long-term contact with irritant red clay soil of volcanic origin.^6^ Podoconiosis was first identified in 1980 as an important cause of tropical lymphoedema, yet still continues to cause significant morbidity.^7,8^

Globally, podoconiosis has been reported in 25 countries in tropical Africa, South East Asia and Latin America, with an estimated 4 million cases.^9,10^ Recently, three countries (Ethiopia, Cameroon and Rwanda) have taken the initiative to map the distribution of podoconiosis. As plans for the mapping of podoconiosis continue to progress, it is imperative to have targets for elimination and a threshold by which to define endemicity. An endemicity threshold will help to identify areas considered endemic for podoconiosis and requiring interventions. This information will be useful for public health policy and planning.

In 2011 WHO identified podoconiosis as one of the neglected tropical diseases (NTDs).^11^ Although this inclusion was an important step forward, to date there is no clear global strategy for the elimination or control of podoconiosis. Communities or countries require a pre-specified threshold to achieve the goal of elimination of podoconiosis as a public health problem. For other NTDs, elimination targets and endemicity classifications are often based on prevalence of the infective organism. For lymphatic filariasis (LF), the target is the reduction of the microfilaraemia rate to below 1% in previously endemic districts.^12,13^ This is not possible with podoconiosis, since the agent is mineral rather than biological, so targets will rather be based on morbidity (lymphoedema) prevalence.

As global interest in podoconiosis increases it is important to have clear goals and indicators to measure progress towards control and elimination. Here we present the results of a Delphi assessment by experts working on podoconiosis and other NTDs. The objectives of this paper are 1) produce case definitions of podoconiosis for surveillance; 2) identify appropriate endemicity classes for podoconiosis (non-endemic, hypo-, meso- and hyper-endemic); 3) identify targets for elimination of podoconiosis from a country; 4) define clinical outcomes to monitor progress.

## Materials and methods

### Description of Delphi techniques

The Delphi survey technique is an iterative email or web-based survey to reach a consensus among a group of experts who are familiar with a subject area.^14^ The approach uses well-designed sequential surveys in which each round depends on the response from the previous round. Each round uses feedback from the previous rounds from the same group of experts. Once the experts are selected, they are asked to provide judgement on the concepts of the subject under consideration using open-ended questions.^15^ The next phase asks the same panelists to rank the items based on a Likert scale, measuring their importance. Following that, several rounds may be needed until a consensus on each item has been achieved based on the results of the quantitative analysis.^16^

### Design and participants

**Figure 1.**
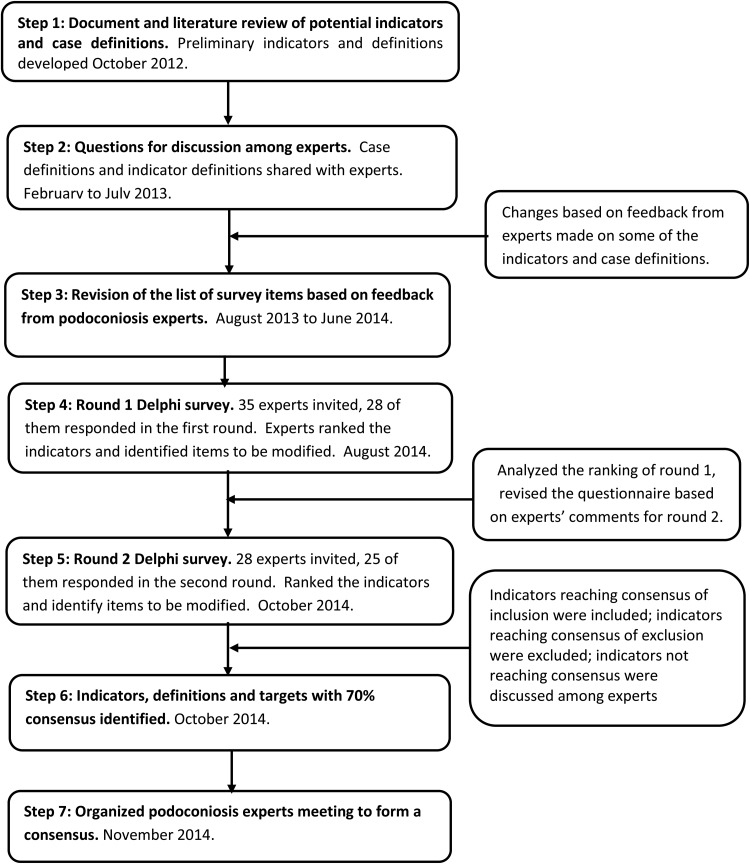
Flowchart of the Delphi process for the development of case definitions, endemicity classifications and elimination targets.

We used a Delphi method to define case definition, endemicity class, elimination targets and clinical outcomes of podoconiosis. We used a four-round Delphi method. In the first two rounds a small group of experts working on podoconiosis were involved in developing the tool. In the subsequent two rounds a wider group of experts working on podoconiosis and other NTDs was involved. These later rounds were conducted from August to November 2014 (Figure 1).

The selection of experts for the Delphi process was done using a purposive sampling technique (expert sampling). For the first two rounds, experts with experience in podoconiosis research and programs were included. For the subsequent two rounds, the following eligibility criteria were used to select the experts: individuals working on NTDs at national or global level; individuals working on podoconiosis research or program management; senior podoconiosis professionals with in-depth knowledge and experience of control; experts involved in developing indicators and targets for other NTDs.

During recruitment, potential experts were approached (initially via e-mail or by telephone) and provided with a detailed explanation of the study and its objectives. A total of 28 national and international experts participated in the Delphi analysis process.

### Questionnaire development

We developed the questionnaire after review of the literature and in consultation with podoconiosis experts. Experiences of developing elimination targets for other diseases were reviewed, and podoconiosis prevalence estimates from affected countries^17–21^ were used to draft the questionnaire. The questionnaire included general information about the purpose of the study and the need for targets for podoconiosis control. A list of possible case definitions, endemicity classifications, elimination targets and monitoring indicators was provided, and respondents were asked to indicate their agreement or disagreement with each item and to provide alternative options for each (Supplementary information and Figure 1).

### Data collection and analysis

The case definitions, endemicity classifications, elimination targets and monitoring indicators were developed between October 2012 and November 2014 in a series of four reiterative surveys. Feedback on each item was received and the questionnaire was modified. In the last two rounds, experts were asked to score items in terms of importance on a 9-point Likert scale for each indicator ranging from 1=not important to 9=extremely important.

For this study and in line with other related studies,^22,23^ we set the consensus level as follows: 1) consensus of inclusion: >70% of participants scored the item ≥7; 2) consensus of exclusion: >70% of participants scored the item ≤5; 3) no consensus: item failed to meet either of the above criteria. The responses in the first-round survey were analyzed using descriptive statistics and the results were sent back to the experts for review and ratification. Items which were recommended for modification by the experts were revised and added to the second round questionnaire; new items suggested by the experts were also added to the second round questionnaire. In the second questionnaire, participants were asked to re-rank the items from the first round. Second round Delphi responses that reached 70% consensus were determined to be appropriate items for assessing different aspects of podoconiosis elimination. The final results were presented to experts for discussion and final consensus, leading to selection of the final targets and indicators.

Median and mode were used to describe the central tendency of expert responses.^15,24,25^ The coefficient of variation (CV) was used to describe the variability of expert responses.^15,23^ The CV is the ratio of the standard deviation of the responses of the experts on a specific item to its corresponding mean (average). Therefore, each survey item in each round yielded one CV value.^16^

## Results

### Characteristics of the experts

During the first round of the Delphi survey, questionnaires were sent to 35 experts, 28 of whom responded. These 28 experts were working at global or national level and were based in six countries. All of the experts had Masters level education or above. Twenty-five experts responded to the second round survey. Descriptive information on the experts is presented in Table 1.

**Table 1.** Characteristics of experts who completed the surveys

Characteristics	First round survey (n=28)	Second round survey (n=25)
Age (years)		
<35	12 (42.9%)	12 (48.0%)
35–45	10 (35.7%)	7 (28.0%)
45–54	2 (7.1%)	2 (8.0%)
55–64	3 (10.7%)	4 (16.0%)
≥65	1 (3.6%)	0
Gender		
Female	8 (28.6%)	7 (28.0%)
Male	20 (71.4%)	18 (72.0%)
Education		
MSc/MA/MPH	17 (60.7%)	13 (52.0%)
MD	4 (14.3%)	4 (16.0%)
PhD	7 (25.0%)	8 (32.0%)
Work unit		
Global	8 (28.6%)	7 (28.0%)
National	20 (71.4%)	18 (72.0%)
Professional level^a^		
Middle	8 (28.6%)	9 (36.0%)
Associate senior	12 (42.8%)	9 (36.0%)
Senior	8 (28.6%)	7 (28.0%)
Main area of expertise		
Expert on podoconiosis	13 (46.4%)	13 (52.0%)
Expert on other NTDs	12 (42.9%)	9 (36.0%)
Expertise on podoconiosis as well as other NTDs	3 (10.7%)	3 (12.0%)

^a^ Middle: assistant researcher/lecturer/program manager; Associate Senior: assistant/associate professor, national program coordinator, clinician specialist; Senior: professor/director/senior scientist.

### Results of the first round Delphi survey

The results of the first round Delphi survey are shown in Table 2. The median score of items shown in Table 2 ranged from 6.5 to 9.0. Most of the items had CV score of less than 0.3 indicating good central tendencies for expert scores, and thus good consensus.

**Table 2.** Results of the first round survey Delphi process for the development of case definitions, endemicity classifications and elimination targets

Survey items	Median^a^	Mode^a^	CV	Consensus (% score for a survey item of >7)^b^
Section 1: case definitions				
Suspected case: any lymphoedema^c^ of the lower limb of any duration (at this stage we do not expect to make a differential diagnosis but need to record the actual numbers of people with lymphoedema, even if a medical diagnosis has not been confirmed).	8.0	9.0	0.2	73.1
Probable case: any lymphoedema^c^ of the lower limb present for more than 3 months in a resident of, or a long-term visitor to, an endemic area.	7.5	8.0	0.3	57.7
Confirmed case: lymphoedema^c^ of the lower limb present for more than 3 months in a resident of, or long term visitor to an endemic area, for which other causes have been excluded (onchocerciasis, LF, leprosy, Milroy syndrome, heart or liver failure, etc).	8.0	9.0	0.1	88.5
Section 2: endemicity classification				
Non-endemic: <1% prevalence among adults ≥15 years old.	7.5	9.0	0.4	76.9
Hypo-endemic: ≥1 to <3% prevalence among adults ≥15 years old.	8.0	9.0	0.3	76.9
Meso-endemic: 3 to <10% prevalence among adults ≥15 years old.	8.0	9.0	0.3	76.9
Hyper-endemic: ≥10% prevalence among adults ≥15 years old.	8.0	9.0	0.3	76.9
Section 3: elimination				
Elimination of podoconiosis from a district:				
Prevalence is <1% (among individuals ≥15 years old) after 10 years of program implementation, AND	8.0	9.0	0.3	73.1
More than 90% of lymphoedema cases are treated adequately after 10 years of program implementation.	6.5	9.0	0.3	50.0
Elimination of podoconiosis from a country:				
Prevalence of untreated podoconiosis is maintained at <1% (among individuals >15 years old) in 100% of sample villages over a 10 year period, AND	7.0	9.0	0.3	61.5
Prevalence of early signs of podoconiosis among children aged 10–15 years after 10 years of control program implementation is <1 in 10 000, AND	8.0	9.0	0.3	70.8
Greater than 95% of the population in endemic districts wear protective shoes, AND	8.5	9.0	0.2	76.9
Greater than 90% of lymphoedema cases are treated adequately.	7.0	9.0	0.3	61.5
Key indicators for the podoconiosis elimination monitoring:				
Prevalence of podoconiosis (%): number of old and new cases of podoconiosis in the implementation unit (individuals aged ≥15 years) divided by total population ≥15 years old in the same area, x 100.	9.0	9.0	0.2	84.6
Case detection rate (%): number of new cases of podoconiosis in the implementation unit divided by total population at risk in the same area, x 100.	9.0	9.0	0.1	88.4
Treatment completion rate (%): number of patients that completed the required duration of treatment divided by all new podoconiosis cases that started treatment in a given period, x 100.	8.5	9.0	0.2	86.9
Coverage of shoe wearing (%)(point prevalence in sampled villages): number of individuals wearing shoes (aged >1 year old) in implementation unit divided by total number of individuals aged >1 year old in the same area, x 100.	9.0	9.0	0.2	84.6
Section 4: monitoring clinical outcomes				
Treatment completion: a patient who has completed the full course of the initial treatment given at health facility/community level.	9.0	9.0	0.2	92.3
Defaulter: a patient who has been on treatment and whose treatment was interrupted for 2 or more consecutive months.	8.0	9.0	0.3	73.1
Treatment success: treatment is successful if an incapacitated patient can assume normal activities following treatment.	8.0	9.0	0.3	69.2
Key indicators for monitoring progress:				
>95% of the population in endemic districts consistently wears protective shoes (measured for the last 1 year).	8.0	9.0	0.2	80.8
>90% of the population in the endemic districts practices proper foot hygiene^d^ (measured for the last 1 year).	8.0	9.0	0.3	65.3

CV: coefficient of variation; LF: lymphatic filariasis.

^a^ A 9-point Likert scale for each indicator ranging from 1=not important to 9=extremely important was used.

^b^ We set the consensus level as follows: 1) consensus of inclusion: >70% of participants scored the item ≥7; 2) consensus of exclusion: >70% of participants scored the item ≤5; 3) no consensus: item failed to meet either of the above criteria.

^c^ Lymphoedema: swelling caused by the collection of fluid in tissue.^44^

^d^ Proper foot hygiene is defined as washing once per day using soap and water over the period of one year.

For case definitions, the median and mode scores were at least 7.5 and 8.0 respectively. All of the indicators in this category had CV scores of 0.3 or less. Apart from the ‘probable case’ definition, the rest reflected at least 70% agreement (≥7) among the experts. The experts suggested that the ‘probable case’ definition should be modified. In the endemicity category, all the items had a median score of at least 7.5 and a mode score of 9.0. In this category at least 70% of experts were in consensus over all the indicators, and apart from the ‘non-endemic’ category, all had CV scores of 0.3 or less.

In the ‘elimination target’ category, of the ten items, nine had median score ≥7.0 and mode score of 9.0. Apart from three items, consensus was found among 70% or more of the experts. Two of the items with less than 70% agreement were related to lymphoedema management. The experts commented that the term ‘adequate treatment’ should be clearly defined.

In the ‘monitoring clinical outcome’ category, all five items had median score of ≥8.0 and mode score of 9.0. Of the five items, three reached consensus among >70% of the experts. The two items with less than 70% agreement were ‘treatment success’ and ‘measure of proper foot hygiene’. In the former, the experts recommended that the term ‘incapacitated’ should be defined. They also indicated that the measurement of foot hygiene is poorly defined, and essentially a subjective measurement.

None of the items fulfilled the criteria of consensus of exclusion, therefore none were deleted from the second round of the survey. In the ‘case definition’ category, experts suggested that 3 months duration of the symptoms was too short due to the chronic nature of podoconiosis lymphoedema, so the case definition was revised to reflect lymphoedema duration of at least 1 year. In addition, the experts recommended that long term visitors should not be included in the case definition since podoconiosis requires an extended period of exposure to red clay soil in order to occur.^9,26^ Long term visits (defined as at least 6 months) would not be sufficient exposure for podoconiosis occurrence. For treatment of lymphoedema, the experts indicated that the 90% target was low and suggested it should be raised to 95% for round 2.

### Results of the second round Delphi survey

All of the case definitions, endemicity classes, elimination targets and clinical outcomes reached >70% consensus among experts (Table 3). The median and mode scores ranged between 8.0 and 9.0, and the CV score was less than 0.3 for all of the indicators and case definitions, demonstrating a high degree of consensus among the experts.

**Table 3.** Results of the second round survey Delphi process for the development of case definitions, endemicity classifications and elimination targets

Survey items	Median^a^	Mode^a^	CV	Consensus (% score for a survey item of >7)^b^
Section 1: case definitions				
Suspected case: any lymphoedema^c^ of the lower limb of any duration (at this stage we do not expect to make a differential diagnosis but need to record the actual numbers of people with lymphoedema, even if a medical diagnosis has not been confirmed).	9.0	9.0	0.1	92.0
Probable case: any lymphoedema^c^ of the lower limb present for more than 1 year in a resident of an endemic area.	8.0	9.0	0.3	76.0
Confirmed case: lymphoedema^c^ of the lower limb present for more than 1 year in a resident of an endemic area, for which other causes have been excluded (onchocerciasis, LF, leprosy, Milroy syndrome, heart or liver failure, etc).	9.0	9.0	0.1	96.0
Section 2: endemicity classification				
Non-endemic: <1% prevalence among adults ≥15 years old.	9.0	9.0	0.3	76.0
Hypo-endemic: ≥1 to <3% prevalence among adults ≥15 years old.	8.0	8.0	0.2	84.0
Meso-endemic: 3 to <10% prevalence among adults ≥15 years old.	8.0	8.0	0.2	84.0
Hyper-endemic: ≥10% prevalence among adults ≥15 years old.	9.0	9.0	0.2	88.0
Section 3: elimination				
Elimination of podoconiosis from a district:				
The prevalence is <1% (among individuals ≥15 years old) after 10 years of program implementation, AND	8.0	9.0	0.2	92.0
More than 95% of lymphoedema cases are treated adequately after 10 years of program implementation.	9.0	9.0	0.1	88.0
Elimination of podoconiosis from a country:				
Prevalence of untreated podoconiosis is maintained at <1% (among individuals >15 years old) in 100% of sample villages over a 10 year period, AND	8.0	9.0	0.2	84.0
Prevalence of early signs of podoconiosis among children aged 10–15 years after 10 years of control program implementation is <1 in 10 000, AND	9.0	9.0	0.2	88.0
Greater than 95% of the population in endemic districts wear protective shoes, AND	9.0	9.0	0.2	84.0
Greater than 95% of lymphoedema cases are treated adequately.	8.0	8.0	0.1	84.0
Key indicators for the podoconiosis elimination monitoring:				
Prevalence of podoconiosis (%): number of old and new cases of podoconiosis in the implementation unit (individuals aged ≥15 years) divided by the total population ≥15 years old in the same area, x 100.	9.0	9.0	0.1	96.0
Case detection rate (%): number of new cases of podoconiosis in the implementation unit divided by the total population at risk in the same area, x 100.	9.0	9.0	0.1	100.0
Treatment completion rate (%): number of patients that completed the required duration of treatment divided by all new podoconiosis cases that started treatment in a given period, x 100.	9.0	9.0	0.1	100.0
Coverage of shoe wearing (%)(point prevalence in sampled villages): number of individuals wearing shoes (aged >1 year old) in implementation unit divided by total number of individuals aged >1 year old in the same area, x 100.	9.0	9.0	0.1	92.0
Section 4: monitoring clinical outcomes				
Treatment completion: a patient who has completed the full course of the initial treatment given at health facility/community level.	9.0	9.0	0.1	96.0
Defaulter: a patient who has been on treatment and whose treatment was interrupted for 2 or more consecutive months.	9.0	9.0	0.2	96.0
Treatment Success: treatment is successful if an incapacitated patient can assume normal activities following treatment.	9.0	9.0	0.2	92.0
Key indicators for monitoring progress:				
>95% of the population in endemic districts consistently wears protective shoes (measured for the last 1 year).	9.0	9.0	0.2	92.0
>90% of the population in the endemic districts practices proper foot hygiene^d^ (measured for the last 1 year).	9.0	9.0	0.1	96.0

CV: coefficient of variation; LF: lymphatic filariasis.

^a^ A 9-point Likert scale for each indicator ranging from 1=not important to 9=extremely important was used.

^b^ We set the consensus level as follows: 1) consensus of inclusion: >70% of participants scored the item ≥7; 2) consensus of exclusion: >70% of participants scored the item ≤5; 3) no consensus: item failed to meet either of the above criteria.

^c^ Lymphoedema: swelling caused by the collection of fluid in tissue.^44^

^d^ Proper foot hygiene is defined as washing once per day using soap and water over the period of one year.

### Case definition of podoconiosis for surveillance

Three levels of case definition were arrived at: 1) suspected case: any lymphoedema of the lower limb of any duration; 2) probable case: any lymphoedema of the lower limb present for more than 1 year in a resident of an endemic area; 3) confirmed case: lymphoedema of the lower limb present for more than 1 year in a resident of an endemic area, for which other causes (e.g. onchocerciasis, LF, leprosy Milroy syndrome, heart or liver failure) have been excluded.

### Endemicity classifications

Percentage prevalences were agreed for four endemicity classifications; ‘non-endemic’, ‘hypo-endemic’, ‘meso-endemic’ and ‘hyper-endemic’ (Table 3), based on knowledge of sub-district prevalence.

### Elimination targets

Consensus was reached on the following elimination targets: Podoconiosis is defined as being eliminated from an endemic district or implementation unit if the prevalence of untreated podoconiosis is less than 1% (among individuals ≥15 years old), and more than 95% of lymphoedema cases are treated adequately after 10 years of program implementation. Podoconiosis is defined as being eliminated from a country when the following four targets are achieved: 1) Prevalence of untreated podoconiosis is maintained at less than 1%(among individuals >15 years old) in 100% of sample villages over a 10-year period. 2) Prevalence of early signs of podoconiosis among children 10 to 15 years after 10 years of control program implementation is less than 1 in 10 000. 3) Greater than 95% of the population in endemic districts wear protective shoes. 4) Greater than 95% of lymphoedema cases are treated adequately. The assumption here is that if the prevalence in an implementation unit is less than 1%, the formal health sector can manage patients reactively, provided the formal health sector is trained and equipped to do so, and prevention activities can be run by the health system, without the need for a control program.

## Discussion

In 2011 WHO defined podoconiosis as one of the NTDs, including it under the ‘other conditions’ category.^11^ While this was a step forward for the control of the disease, no clear targets or endemicity classifications exist. Such targets and definitions are important to consolidate efforts to control podoconiosis. Historically, podoconiosis appears to have been eliminated from northern Africa due to socioeconomic development and widespread shoe-wearing practices,^7^ suggesting that podoconiosis elimination is within our reach.

Using a Delphi assessment process, we defined the endemicity threshold for podoconiosis to be ≥1%, the underlying assumption being that in the absence of point-of-care diagnosis overestimation of cases may occur. In areas where there is no podoconiosis or LF, the underlying prevalence of lymphoedema is approximately 0.1–0.5%.^27^ Hence a threshold of ≥1% will help take into account such residual lymphoedema cases. The second rationale for such a cut-off point is that even if the 1% of cases are confirmed to be due to podoconiosis, the local health system can respond without the need for establishing a control program. Nonetheless, even in such cases, the health providers working in the system should be given clear training and be equipped with the necessary supplies.

We also generated endemicity classifications for podoconiosis. Evidence from national and small-scale surveys indicates that the prevalence of podoconiosis ranges from 1–10% with few prevalence estimates >10% per district.^17–19, 21^ Three endemicity classes will allow areas within the highest category to be prioritized and will enable monitoring of progress within these areas. These thresholds are intended more to prioritize endemic implementation units (administrative units used as the basis for making decisions about morbidity management), than to indicate any particular biological consequences of disease prevalence in contrast to infectious diseases.^28^

We have also produced targets for monitoring elimination of podoconiosis. Since there is no biological cause of podoconiosis, we cannot aim for interruption of transmission; the focus will be on access to preventive and treatment services. The combination of these elimination targets is intended to avoid the debates such as those surrounding the leprosy elimination targets.^29^ Our targets address access to morbidity management for the backlog of cases as well as prevention of new disease (monitored through shoe-wearing practice and identification of new cases among children aged between 10–15 years). We suggest a 10 year time frame for the evaluation of control and elimination programs, given the challenge of addressing the backlog of cases and addressing behavioral change with regard to preventive behaviors such as shoe wearing and regular foot hygiene practice. This will also enable progress in reducing incident cases to be monitored. Table 4 summarises the sources of data from which measures of key indicators are likely to be derived. These include routine sources (health management information systems and clinical records) and specific patient and community surveys.

**Table 4.** Sources of data

Indicators	Measure	Data source
Section 1: case definition		
Suspected cases	Number of suspected cases of podoconiosis	Clinical record or HMIS
Probable cases	Number of probable cases of podoconiosis	Clinical record or HMIS
Confirmed cases	Number of confirmed cases of podoconiosis	Clinical record or HMIS
Section 2: endemicity classification		
Non-endemic IUs	Number of non-endemic IUs	Mapping and evaluation surveys
Hypo-endemic IUs	Number of hypo-endemic IUs	Mapping and evaluation surveys
Meso-endemic IUs	Number of meso-endemic IUs	Mapping and evaluation surveys
Hyper-endemic IUs	Number of hyper-endemic IUs	Mapping and evaluation surveys
Section 3: elimination		
Elimination of podoconiosis from an IU:		
Prevalence of untreated podoconiosis (among individuals aged ≥15 years) in an endemic IU	Prevalence of podoconiosis	Mapping and evaluation surveys
Percent of lymphoedema cases treated adequately	Percentage cases of lymphoedema treated adequately	Evaluation surveys
Prevalence of early signs of podoconiosis among children aged 10–15 years in an endemic IU	Prevalence of early signs of podoconiosis	Mapping and evaluation surveys
Proportion of the population in an endemic IU wearing protective shoes	Proportion of individuals wearing shoes	Mapping and evaluation surveys
Key indicators for the podoconiosis elimination monitoring:		
Case detection rate: proportion of patients newly diagnosed withpodoconiosis in the IU	Proportion of new cases of podoconiosis in the IU	Community-based survey and clinical record or HMIS
Treatment completion rate: proportion of patients that completed the required duration of treatment divided by all patients newly diagnosed with podoconiosis that started treatment in a given period	Proportion of patients that completed the required duration of treatment	Podoconiosis patient survey or clinical record or HMIS
Section 4: monitoring clinical outcomes		
Defaulter rate: percentage of patients with default treatment	Percentage of patients who experienced default treatment	Podoconiosis patient survey and clinical record review or HMIS
Treatment success 1: defined as the proportion of advanced stage patients (podoconiosis clinical stage 3, 4 or 5)^45^ who can resume normal activities following treatment, maintained over a follow-up period of 1 year.	Proportion of advanced stage podoconiosis patients who resumed normal activities, maintained for a 1 year period	Podoconiosis patient survey and clinical record review or HMIS
Treatment success 2: defined as the proportion of early stage patients (clinical stage 1 or 2)^45^ with one stage decrease after completion of treatment, maintained for over a period of 1 year.	Proportion of early stage podoconiosis patients who decreased one stage after completion of treatment, maintained for 1 year	Podoconiosis patient survey and clinical record review or HMIS
Key indicators for monitoring progress:		
Proportion of the population in the endemic districts that practice proper foot hygiene (measured for the last 1 year)	Proportion of individuals who practice proper foot hygiene	Community-based survey

HMIS: health management information system; IU: implementation unit (administrative units used as the basis for making decisions about morbidity management).

For the first time, we have developed a case definition for podoconiosis which can be used within the routine surveillance system. The sensitivity and specificity of our definition must be evaluated in future studies. These broad definitions will capture individuals with lymphoedema who may require foot care services. With global movement towards integrated foot care services,^30^ identifying the specific causes of lymphoedema may not be necessary. While the definition here is sufficient for surveillance purposes, if identification of the specific cause of lymphoedema is necessary, confirmatory tests to exclude other potential causes of lymphoedema will be required until podoconiosis-specific point-of-care diagnostic tools are developed. Other causes of lymphoedema are excluded on the basis of history and physical examination.^18,31^ The diagnosis of podoconiosis is based on a history including details of where in the lower limb the swelling started from, the age at which swelling started and the presence of a family member with similar conditions.^31,32^ Clinical examination for intact sensation in the lower leg, and the absence of swelling in the groin area, is also important. In addition, podoconiosis patients must be negative for all tests for LF.^31^

Measuring clinical outcomes of indivduals with podoconiosis is a complex phenomenon. Although simple lymphoedema management for people with podoconiosis exists (i.e. foot hygiene, foot and skin care, wound care, compression, exercises and elevation, treatment of acute attack and use of shoes and socks to reduce further exposure to the irritant soil),^33–35^ it has not been properly evaluated or standardized. The duration of treatment and follow-up frequency are areas currently under research. Therefore, only three areas of clinical outcome (treatment completion, defaulter and treatment success) are considered in the ‘monitoring clinical outcomes’ definition (Table 3). Other areas of clinical outcome will be defined once the ongoing randomized controlled trial^36^ is completed. Currently, a patient is regarded as having completed treatment when he/she has followed supervised morbidity management for at least 3 months. Within these 3 months, if a patient misses two consecutive monthly clinical appointments, they are considered a defaulter. These end points are important in monitoring the success of morbidity management programs.

The interventions for both prevention and treatment of podoconiosis are simple,^9,34^ and short term goals such as restored function and improved quality of life can be achieved among lymphoedema cases after just 3 months of treatment.^33,34^ Promotion of shoe wearing for podoconiosis prevention may have multiple health benefits.^37^ Integrated morbidity management in the context of foot care may become an important approach to avoid duplication of efforts and to utilize the available resources efficiently. It is possible to integrate foot care services for podoconiosis with those for Buruli ulcer, LF and leprosy.^30^ Currently the morbidity management services are provided by very few faith-based organizations and NGOs in endemic countries.^33,34^ Provision of free-of-charge or low-cost prevention and treatment through government programmes will be critical. People with podoconiosis are often poor^38^ and marginalized due to their condition, and introduction of user fees might hinder their access to the services.

Prevention of podoconiosis is an important component of its elimination. Promotion of shoe wearing as a health intervention should be advocated for.^37^ For those individuals who cannot afford shoes themselves, subsidized shoe distribution should be considered. Continued research should focus on identifying point-of-care diagnostic tools for podoconiosis to ascertain cases and verify elimination. At the start of elimination programs, definitive diagnosis may not be a priority, but as the ‘end game’ approaches, robust, sensitive and specific diagnostic tests will be required. In the earlier phases of elimination, a syndromic approach may suffice for the diagnosis of podoconiosis.^31^ In the long term, identification of biomarkers of podoconiosis will be vital.

With all these important findings our study is not without limitations. First, research into and intervention against podoconiosis are currently present in only a few endemic countries, so there are few experts in podoconiosis. Nonetheless, we have included almost all the top experts in podoconiosis research and programing working in endemic counties globally. Second, there are many important questions unanswered so far about podoconiosis, such as treatment duration and outcomes. This means that there is a need to define these indicators once appropriate evidence is generated. Third, full operationalisation of several components of the case definitions and elimination targets is still in progress. Operational definition of ‘adequate treatment’ of lymphoedema is an issue being explored in relation to lymphatic filariasis as well as podoconiosis. Similarly ‘practicing proper foot hygiene’ and ‘wearing protective footwear’ will both require robust operational definitions.

### Conclusions

Untreated lymphoedema due to podoconiosis causes significant deformity, disability, social and productivity loss in endemic countries.^38–40^ The disabilities associated with podoconiosis are preventable with simple preventive measures (consistent shoe wearing and foot hygiene) and early lymphoedema morbidity management.^18,34^ These simple interventions can be integrated into the health system and delivered at low cost.^41^ It is time to capitalize on the experience of some endemic countries and provide the intervention sustainably and at scale, with clear aims and targets.^33,35,42,43^ Defining elimination targets and endemicity classification is critical for mobilizing communities and stakeholders and ensuring accountability in relation to elimination of podoconiosis. The indicators described here may serve as a starting point towards a global strategy for podoconiosis elimination.

## Supplementary data

Supplementary data are available at International Health online (http://inthealth.oxfordjournals.org/).

Supplementary Data

## References

[1] Heymanna DL Control, elimination, eradication and re-emergence of infectious diseases: getting the message right. Bull World Health Organ 2006;84:82. 16501719 10.2471/blt.05.029512 PMC2626526

[2] Whitty CJ Milroy Lecture: eradication of disease: hype, hope and reality. Clin Med 2014;14:419–21. 25099846 10.7861/clinmedicine.14-4-419 PMC4952838

[3] Centers for Disease Control and Prevention. Global disease elimination and eradication as public health strategies. Proceedings of a conference. Atlanta, Georgia, USA. 23–25 February 1998. MMWR 1999;48 (Suppl):1–208. 11186140

[4] Dowdle WR The principles of disease elimination and eradication. Bull World Health Organ 1998;76 (Suppl 2):22–5. 10063669 PMC2305684

[5] Uniting to combat neglected tropical diseases. Ending the neglect & reaching 2020 goals. London declaration on neglected tropical diseases; 2012 http://unitingtocombatntds.org/sites/default/files/resource_file/london_declaration_on_ntds.pdf [accessed 2 May 2015].

[6] Davey G, Newport M Podoconiosis: the most neglected tropical disease? Lancet 2007;369:888–9. 17368134 10.1016/S0140-6736(07)60425-5

[7] Price EW Podoconiosis: Non-filarial elephantiasis. Oxford: Oxford Medical Publications; 1990.

[8] Price EW Non-filarial elephantiasis - confirmed as a geochemical disease, and renamed podoconiosis. Ethiop Med J 1988;26:151–3. 2843362

[9] Davey G, Tekola F, Newport MJ Podoconiosis: non-infectious geochemical elephantiasis. Trans R Soc Trop Med Hyg 2007;101:1175–80. 17976670 10.1016/j.trstmh.2007.08.013

[10] Tekola Ayele F, Adeyemo A, Finan C et al HLA class II locus and susceptibility to podoconiosis. N Engl J Med 2012;366:1200–8. 22455414 10.1056/NEJMoa1108448 PMC3350841

[11] Davey G, Bockarie M, Wanji SAD et al Launch of the international podoconiosis initiative. Lancet 2012;379:1004. 22423883 10.1016/S0140-6736(12)60427-9

[12] Rebollo MP, Bockarie MJ Toward the elimination of lymphatic filariasis by 2020: treatment update and impact assessment for the endgame. Expert Rev Anti Infect Ther 2013;11:723–31. 23879610 10.1586/14787210.2013.811841

[13] WHO. Global program to eliminate lymphatic filariasis. Wkly Epidemiol Rec 2006;81:221–232. 16749186

[14] Mullen PM Delphi: myths and reality. J Health Organ Manag 2003;17:37–52. 12800279 10.1108/14777260310469319

[15] Kalaian SA, Kasim RM Terminating sequential Delphi survey data collection. Pract Assess Res Eval 2012;17:1–10.

[16] Keeney S, Hasson F, McKenna HP A critical review of the Delphi technique as a research methodology for nursing. Int J Nurs Stud 2001;38:195–200. 11223060 10.1016/s0020-7489(00)00044-4

[17] Deribe K, Brooker SJ, Pullan RL et al Spatial distribution of podoconiosis in relation to environmental factors in Ethiopia: a historical review. PLoS ONE 2013;8:e68330. 23874587 10.1371/journal.pone.0068330 PMC3706425

[18] Deribe K, Brooker SJ, Pullan RL et al Epidemiology and individual, household and geographical risk factors of podoconiosis in Ethiopia: results from the first nationwide mapping. Am J Trop Med Hyg 2015:148–58. 25404069 10.4269/ajtmh.14-0446 PMC4288951

[19] Onapa AW, Simonsen PE, Pedersen EM Non-filarial elephantiasis in the Mt. Elgon area (Kapchorwa District) of Uganda. Acta Trop 2001;78:171–6. 11230827 10.1016/s0001-706x(00)00185-6

[20] Tekola Ayele F, Alemu G, Davey G, Ahrens C Community-based survey of podoconiosis in Bedele Zuria woreda, southwest Ethiopia. Int Health 2013;5:119–25. 24030111 10.1093/inthealth/iht003 PMC3889643

[21] Wanji S, Tendongfor N, Esum M et al Elephantiasis of non-filarial origin (podoconiosis) in the highlands of north-western Cameroon. Ann Trop Med Parasitol 2008;102:529–40. 18782492 10.1179/136485908X311849

[22] Cantrill JA, Sibbald B, Buetow S Indicators of the appropriateness of long-term prescribing in general practice in the United Kingdom: consensus development, face and content validity, feasibility, and reliability. Qual Health Care 1998;7:130–5. 10185138 10.1136/qshc.7.3.130 PMC2483608

[23] Li Y, Ehiri J, Hu D et al Framework of behavioral indicators for outcome evaluation of TB health promotion: a Delphi study of TB suspects and TB patients. BMC Infect Dis 2014;14:268. 24884569 10.1186/1471-2334-14-268 PMC4030006

[24] Hasson F, Keeney S, McKenna H Research guidelines for the Delphi survey technique. J Adv Nurs 2000;32:1008–15. 11095242

[25] Hsu CC, Sandford BA The Delphi technique: making sense of consensus. Pract Assess Res Eval 2007;12:1–8.

[26] Kloos H, Bedri Kello A, Addus A Podoconiosis (endemic non-filarial elephantiasis) in two resettlement schemes in western Ethiopia. Trop Doc 1992;22:109–12. 10.1177/004947559202200306 1641880

[27] Moffatt CJ, Franks PJ, Doherty DC et al Lymphoedema: an underestimated health problem. QJM 2003;96:731–8. 14500859 10.1093/qjmed/hcg126

[28] Hay SI, Smith DL, Snow RW Measuring malaria endemicity from intense to interrupted transmission. Lancet Infect Dis 2008;8:369–78. 18387849 10.1016/S1473-3099(08)70069-0 PMC2653619

[29] Lockwood DN, Shetty V, Penna GO Hazards of setting targets to eliminate disease: lessons from the leprosy elimination campaign. BMJ 2014;348 (g1136). 10.1136/bmj.g1136 24508610

[30] Ichimori K, King JD, Engels D et al Global programme to eliminate lymphatic filariasis: the processes underlying programme success. PLoS Negl Trop Dis 2014;8:e3328. 25502758 10.1371/journal.pntd.0003328 PMC4263400

[31] Sime H, Deribe K, Assefa A et al Integrated mapping of lymphatic filariasis and podoconiosis: lessons learnt from Ethiopia. Parasit Vectors 2014;7:397. 25164687 10.1186/1756-3305-7-397 PMC4153915

[32] Tekola-Ayele F, Embiale WY Podoconiosis: tropical lymphedema of the lower legs. In: Dermatology and Allergology - Principles and Practice. 1st ed. Hong Kong: iConcept Press Ltd; 2014.

[33] Davey G, Burridge E Community-based control of a neglected tropical disease: the mossy foot treatment and prevention association. PLoS Negl Trop Dis 2009;3:e424. 19479039 10.1371/journal.pntd.0000424 PMC2682702

[34] Sikorski C, Ashine M, Zeleke Z, Davey G Effectiveness of a simple lymphoedema treatment regimen in podoconiosis management in southern Ethiopia: one year follow-up. PLoS Negl Trop Dis 2010;4:e902. 21152059 10.1371/journal.pntd.0000902 PMC2994920

[35] Tomczyk S, Tamiru A, Davey G Addressing the neglected tropical disease podoconiosis in northern Ethiopia: lessons learned from a new community podoconiosis program. PLoS Negl Trop Dis 2012;6:e1560. 22428078 10.1371/journal.pntd.0001560 PMC3302806

[36] Lang T, Clarke M, Newport M et al A research methodology study to map the process of initiating and operating a randomised controlled trial of podoconiosis treatment in northern Ethiopia. Trials 2013;14 (Suppl 1):O31.

[37] Tomczyk S, Deribe D, Brooker SJ et al Association between footwear use and neglected tropical diseases: a systematic review and meta-analysis. PLoS Negl Trop Dis 2014;8:e3285. 25393620 10.1371/journal.pntd.0003285 PMC4230915

[38] Tekola F, Mariam DH, Davey G Economic costs of endemic non-filarial elephantiasis in Wolaita Zone, Ethiopia. Trop Med Int Health 2006;11:1136–44. 16827714 10.1111/j.1365-3156.2006.01658.x

[39] Gebrehanna E The social burden of podoconiosis in Wolaita zone [MPH thesis]. Addis Ababa: Addis Ababa University; 2005.

[40] Yakob B, Deribe K, Davey G High levels of misconceptions and stigma in a community highly endemic for podoconiosis in southern Ethiopia. Trans R Soc Trop Med Hyg 2008;102:439–44. 18339411 10.1016/j.trstmh.2008.01.023

[41] Tamiru A, Tsegay G, Wubie M et al Podoconiosis patients' willingness to pay for treatment services in northwest Ethiopia: potential for cost recovery. BMC Public Health 2014;19:259. 24642085 10.1186/1471-2458-14-259 PMC4234032

[42] Deribe K, Meribo K, Gebre T et al The burden of neglected tropical diseases in Ethiopia, and opportunities for integrated control and elimination. Parasit Vectors 2012;5:240. 23095679 10.1186/1756-3305-5-240 PMC3551690

[43] Deribe K, Tomczyk S, Tekola-Ayele F Ten years of podoconiosis research in Ethiopia. PLoS Negl Trop Dis 2013;7:e2301. 24130908 10.1371/journal.pntd.0002301 PMC3794913

[44] WHO. Lymphatic filariasis: managing morbidity and preventing disability. Geneva, Switzerland; World Health Organization; Geneva, Switzerland 2013 http://apps.who.int/iris/bitstream/10665/85347/1/9789241505291_eng.pdf [accessed 20 March 2015].

[45] Tekola F, Ayele Z, Mariam DH et al Development and testing of a de novo clinical staging system for podoconiosis (endemic non-filarial elephantiasis). Trop Med Int Health 2008;13:1277–83. 18721188 10.1111/j.1365-3156.2008.02133.x PMC2992944

