# Supplementary material for: Measuring elimination of podoconiosis, endemicity classifications, case definition and targets: an international Delphi exercise
Source: Int Health. 2015 Jul 16;7(5):306–16. doi: 10.1093/inthealth/ihv043 (PMC4550552; doi:10.1093/inthealth/ihv043)
Supplement: Supplementary Data [file supp_7_5_306__index.html]

Measuring elimination of podoconiosis, endemicity classifications, case definition and targets: an international Delphi exercise — Supplementary Data 

# Measuring elimination of podoconiosis, endemicity classifications, case definition and targets: an international Delphi exercise

## Supplementary Data

Supplementary Data

- Supplementary Data - Docx file
