# Supplementary material for: Measuring elimination of podoconiosis, endemicity classifications, case definition and targets: an international Delphi exercise
Source: Int Health. 2015 Jul 16;7(5):306–16. doi: 10.1093/inthealth/ihv043 (PMC4550552; doi:10.1093/inthealth/ihv043)
Supplement: Supplementary Data [file supp_ihv043_ihv043supp.docx]

**Supplementary Information:**

**Measuring elimination of podoconiosis, endemicity classifications, case definition and targets: an international Delphi exercise**

**Defining indicators exercise:**

**Defining elimination targets and endemicity classification of podoconiosis - second round**

**Instructions: how to complete the questionnaire**

Podoconiosis (endemic non-filarial elephantiasis) is a non-infectious geochemical disease often found in barefoot subsistence farmers who are in long-term contact with irritant red clay soil of volcanic origins. The disease causes progressive bilateral swelling of the lower legs.^^[[1]](#footnote-1)^^

As plans for the mapping of podoconiosis continue to progress, it is imperative to have targets for elimination and a threshold for endemicity. An endemicity threshold would help to identify areas which are endemic for podoconiosis and require interventions. This information would be useful for public health policy and planning. For example, districts/villages with a lymphedema prevalence of greater than 1% could be classified as endemic for podoconiosis. This could be further classified using endemicity classes (i.e. hypo-, meso- and hyper-) to show which areas may need high priority. Although residents of areas classified as hypo-endemic might not have podoconiosis treatment sites, these individuals would still be eligible for individual health facility-based treatment if they are diagnosed with podoconiosis.

The vision of the International Podoconiosis Initiative is to eliminate podoconiosis (‘world free of podoconiosis in our lifetimes’). Therefore, this goal needs to have a measurable indicator. Communities or countries should have a pre-specified threshold to achieve the goal of elimination of podoconiosis as a public health problem. For other NTDs, elimination targets and endemicity classifications are often based on prevalence of the infective organism. This is not possible with podoconiosis, so targets will rather be based on morbidity (lymphedema) prevalence. For example, the target of global elimination of leprosy as a public health problem is the reduction of cases to less than 1 per 10 000 population. For lymphatic filariasis (LF), the target is the reduction of microfilaraemia rate to below 1% in previously endemic districts. Similarly, there is a clear need for measurable targets for the elimination of podoconiosis.

**Assumptions:** The prevalence of lymphedema in areas where LF and podoconiosis are not endemic ranges from 1–5 per 1000. Therefore the cut-off is reduced to 1% to accommodate these figures in case of any misclassification. In a district were the prevalence is <1% the cases will be treated in the formal health sector without the need for additional control programs.

The prevalence of podoconiosis ranges between 0–10% with some areas having prevalence >10% based on studies from Ethiopia, Rwanda, Burundi, Cameroon and Uganda.

The current treatment practice is lymphedema management of 3 months. But there is an ongoing RCT which determines adequate treatment of podoconiosis. Therefore adequate will be defined upon completion of the study.

Population at risk is defined as the total number of individuals living in an endemic district.

**Methods:**

Objectives:

The objectives are as follows:

- To decide on a threshold by which an area is called endemic for podoconiosis.
- To identify appropriate endemicity classes for podoconiosis (non-endemic, hypo-, meso- and hyper-).
- To identify the target for elimination of podoconiosis from a country.

We are aiming to reach agreement among a range of professional groups on the key components of endemicity classification and elimination target. We need your help in identifying the issues that are of importance. Please now score the items listed below. The questionnaire is divided into four sections:

**SECTION 1:** Case definition

**SECTION 2:** Endemicity classification

**SECTION 3:** Elimination targets

**SECTION 4:** Monitoring indicators

You are asked to score the importance of potential issues using the following grading:

**A 9-point scale with the anchors ‘‘not important’’ at 1 and ‘‘extremely important’’ at 9**

Please **circle the most appropriate score** in the **column next to each statement.**

Rest assured we will treat your responses in confidence.

**We would be grateful if you could complete the form as thoroughly as possible, but you are not obliged to complete all sections.**

If you have any comments, please use the space provided.

**Scoring: A 9-point scale with the anchors ‘‘not important’’ at 1 and ‘‘extremely important’’ at 9**

| **SECTION 1**  **Statement: *‘‘The following definition is important to develop case definition for podoconiosis surveillance -----*** | | | | |
| --- | --- | --- | --- | --- |
|  | Median score Round 1 | Mode score Round 1 | % agreement Round 1 for scores  7,8 & 9 | **Your score Round 2** |
| **Suspected case:** any lymphoedema of the lower limb of any duration. (At this stage we do not expect to make a differential diagnosis but need to record the actual numbers of people with lymphoedema, even if a medical diagnosis has not been confirmed.) | 8.0 | 9.0 | 73.1% | **1 2 3 4 5 6 7 8 9** |
| **Probable case:** Any lymphoedema of the lower limb present for more than one year in a resident of, or a long-term visitor to, an endemic area. | 7.5 | 8 | 57.7% | **1 2 3 4 5 6 7 8 9** |
| **Confirmed case:** Lymphoedema of the lower limb present for more than one year in a resident of, or long term visitor to an endemic area, for which other causes have been excluded (onchocerciasis, LF, leprosy, Milroy syndrome, heart or liver failure, etc). | 8.0 | 9 | 88.5% | **1 2 3 4 5 6 7 8 9** |
| **SECTION 2**  **Statement *“ The following endemicity classification for podoconiosis is important for prioritization*** | | | | |
| **Non-endemic** <1% prevalence among adults ≥ 15 years old  AND | 7.5 | 9.0 | 76.9% | 1 2 3 4 5 6 7 8 9 |
| **Hypo-endemic** ≥1to <3% prevalence among adults ≥ 15 years old  AND | 8.0 | 9.0 | 76.9% | 1 2 3 4 5 6 7 8 9 |
| **Meso-endemic** 3 to<10% prevalence among adults ≥ 15 years old  AND | 8.0 | 9.0 | 76.9% | 1 2 3 4 5 6 7 8 9 |
| **Hyper-endemic** ≥ 10% prevalence among adults ≥ 15 years old | 8.0 | 9.0 | 76.9% | 1 2 3 4 5 6 7 8 9 |
| **SECTION 3**  **Statement *“ Podoconiosis is eliminated from a district if …*** | | | | |
| The prevalence of untreated podoconiosis is less than 1 percent (among individuals ≥15 years old after 10 years of program implementation.  AND | 8.0 | 9.0 | 73.1% | **1 2 3 4 5 6 7 8 9** |
| More than 95 percent of lymphoedema cases are treated adequately after 10 years of program implementation. | 6.5 | 9.0 | 50.0% | **1 2 3 4 5 6 7 8 9** |
| *Statement “Podoconiosis is eliminated from a* ***country*** *when* | | | | |
| Prevalence of untreated podoconiosis is maintained at less than 1 percent (among individuals >15 years old) in 100 percent of sample villages over a 10 year period. | 7.0 | 9.0 | 61.5% | **1 2 3 4 5 6 7 8 9** |
| Prevalence of early signs of podoconiosis among children 10-15 years after 10 years of control program implementation is less than 1 in 10,000. | 8.0 | 9.0 | 70.8% | **1 2 3 4 5 6 7 8 9** |
| Greater than 95 percent of population in endemic districts wears protective shoes. | 8.5 | 9.0 | 76.9% | **1 2 3 4 5 6 7 8 9** |
| Greater than 90 percent of lymphoedema cases are treated adequately. | 7.0 | 9.0 | 61.5% | **1 2 3 4 5 6 7 8 9** |
| Statement “ *The following key indicators for the podoconiosis elimination are important* *to be monitored* | | | | |
| **Prevalence of podoconiosis (%) =** Number of old and new cases of podoconiosis in the implementation unit (≥15 Years) divided by total population of ≥15 years old in the same area times 100 | 9.0 | 9.0 | 84.6% | **1 2 3 4 5 6 7 8 9** |
| **Case detection rate (%)=** Number of new cases of podoconiosis in the implementation unit in a year divided by total population at risk in the same area times 100 | 9.0 | 9.0 | 88.4% | **1 2 3 4 5 6 7 8 9** |
| **Treatment completion rate** (%)= Number of patients that took completed the required duration of treatment divided by all new podoconiosis cases that started treatment in a given period times 100 | 8.5 | 9.0 | 86.9% | **1 2 3 4 5 6 7 8 9** |
| **Coverage of shoe wearing (%)(point prevalence in sampled villages)=** Number of individuals wearing shoes (>1 years old) in implementation unit divided by total number of individuals >1 years old in the same area times 100 | 9.0 | 9.0 | 84.6% | **1 2 3 4 5 6 7 8 9** |
| **SECTION 4:**  Statement *“ The following key indicators are important for monitoring clinical outcomes* | | | | |
| **Treatment completion:** A patient who has completed the full course of the initial treatment given at health facility/community level. | 9.0 | 9.0 | 92.3% | **1 2 3 4 5 6 7 8 9** |
| **Defaulter:** A patient who has been on treatment and whose treatment was interrupted for 2 or more consecutive months. | 8.0 | 9.0 | 73.1% | **1 2 3 4 5 6 7 8 9** |
| **Treatment Success:** Treatment is successful if an incapacitated patient can assume normal activities following treatment. | 8.0 | 9.0 | 69.2% | **1 2 3 4 5 6 7 8 9** |
| Statement *“ The following key indicators are important for monitoring progress* | | | | |
| > 95% of population in endemic districts consistently wears protective shoes (measured for the last one year). | 8.0 | 9.0 | 80.8% | **1 2 3 4 5 6 7 8 9** |
| >90% of the population in the endemic districts practices proper foot hygiene* (measured for the last one year).(proper foot hygiene is defined as washing once per day using soap and water over the period of one year) | 8.0 | 9.0 | 65.3% | **1 2 3 4 5 6 7 8 9** |

**We are very grateful** **for your help with this defining indicators exercise.**

**References:**

1. Davey G, Tekola F, Newport MJ. Podoconiosis: non-infectious geochemical elephantiasis. Trans R Soc Trop Med Hyg 2007;101:1175–80.

1. [↑](#footnote-ref-1)
